# The neural substrates of neurological soft signs in schizophrenia: a systematic review

**DOI:** 10.1038/s41537-022-00245-9

**Published:** 2022-04-26

**Authors:** Genelle D. Samson, Adrienne C. Lahti, Nina V. Kraguljac

**Affiliations:** 1 Department of Psychology, University of Alabama at Birmingham, Birmingham, AL USA; 2 Department of Psychiatry and Behavioral Neurobiology, University of Alabama at Birmingham, Birmingham, AL USA

**Keywords:** Schizophrenia, Biomarkers

## Abstract

Neurological soft signs (NSS) are common in patients with schizophrenia. However, the neural substrates of NSS remain poorly understood. Using legacy PubMed, we performed a systematic review and included studies that assessed NSS and obtained neuroimaging data in patients with a schizophrenia spectrum disorder published up to June 2020. We systematically reviewed 35 relevant articles. Studies consistently implicate the basal ganglia and cerebellum as structural substrates of NSS and suggest that somatomotor and somatosensory regions as well as areas involved in visual processing and spatial orientation may underlie NSS in psychosis spectrum disorders. Additionally, dysfunction of frontoparietal and cerebellar networks has been implicated in the pathophysiology of NSS. The current literature outlines several structural and functional brain signatures that are relevant for NSS in schizophrenia spectrum disorder. The majority of studies assessed gray matter structure, but only a few studies leveraged other imaging methods such as diffusion weighted imaging, or molecular imaging. Due to this, it remains unclear if white matter integrity deficits or neurometabolic alterations contribute to NSS in the illness. While a substantial portion of the literature has been conducted in patients in the early illness stages, mitigating confounds of illness chronicity, few studies have been conducted in antipsychotic medication-naïve patients, which is a clear limitation. Furthermore, only little is known about the temporal evolution of NSS and associated brain signatures. Future studies addressing these pivotal gaps in our mechanistic understanding of NSS will be important.

## Introduction

Neurological soft signs (NSSs) are minor abnormalities in motor coordination, sensory integration, sequencing of complex motor movements, and the disinhibition of those movements^1^. “Soft” signs are conceptualized as subclinical, non-localized signs of neurological abnormalities, whereas “hard” signs typically refer to impairments in basic sensory, motor, and reflex behaviors which can usually be directly mapped to a specific brain region^2^. This dichotomy largely exists because of the difficulty in attributing those soft signs to specific brain abnormalities; however, as research and technology advance, that narrative is shifting.

NSSs occur in the majority of patients with schizophrenia^2^. They are present more frequently than in healthy subjects and have been suggested to represent a core feature of the illness^3,4^; meta-analyses find that up to 73% of schizophrenia patients perform outside the range of healthy participants on aggregate NSS measures^1^. Interestingly, NSSs are associated with psychosis proneness within the general population^5^, and motor abnormalities in adolescents at risk for psychotic disorders may predict subsequent conversion to psychosis^6–8^. NSSs are significantly more common in people with schizophrenia than in their first-degree relatives, and are more common in first-degree relatives than in healthy controls^9^. While some studies report that NSS scores are diagnostically nonspecific across psychosis spectrum disorders^10,11^ others found differences in NSS severity between patients with schizophrenia and bipolar disorder^12,13^.

Studies measuring NSS suggest an abnormal developmental trajectory of NSS in schizophrenia patients^14,15^. In healthy controls, the relationship between NSS and age tends to be a U-shaped pattern, whereas in schizophrenia patients, the relationship is parallel, with a flat but overall elevated pattern^14^.

The presence of NSSs has been found to be independent of demographic variables and most medication variables^2^, supporting the idea that NSSs are related to the pathophysiology and pointing towards a neurodevelopmental component in the pathophysiology^2^. However, others found NSSs to vary over the clinical course of the disorder^16^. It appears that NSSs may decrease with remission of psychotic symptoms, especially in patients with a remitting chronic course, but not to levels typically seen in healthy controls^16^ suggesting potential clinical utility for these measures in monitoring of disease progression.

Currently, NSSs are not generally assessed in clinical practice^8^ and there is not a universally accepted assessment tool for NSS. Different existing instruments include various items and factor levels^17–22^. The most common instruments include the Neurological Evaluation Scale (NES) which includes 28 items that fall into one of three functional areas of interest (*integrative sensory dysfunction*, *motor incoordination*, and *impaired sequencing of complex motor acts*) or an “Other” domain^17^; the Heidelberg Scale with 17 items which fall into one of five factors (*motor coordination*, *integrative functions*, *complex motor tasks*, *right/left and spatial orientation*, and *hard signs*)^22^; and the Cambridge Neurological Inventory (CNI) with eight categories (*hard neurological signs*, *motor coordination*, *sensory integration*, *primitive reflexes*, *tardive dyskinesia*, *catatonic signs*, *parkinsonism*, and *failure to suppress inappropriate response*)^18^. The different scales and individual item scores are not directly comparable, but subscales, especially with respect to motor sequencing, motor coordination and sensory integration, are contrastable^23^.

In 2014, an activation likelihood estimation (ALE) meta-analysis of six structural and fifteen functional neuroimaging studies reported that that NSS patients with schizophrenia and related psychotic disorders were associated with structural and functional abnormalities of brain regions of the cerebello-thalamo-prefrontal network^24^. Notably, the majority of studies included in this analysis used a go/no-go task as a proxy for motor disinhibition rather than using direct assessments that capture NSS across domains. Building on these efforts, we performed a systematic review of the neuroimaging studies that comprehensively characterized NSS with instruments designed to assess NSS across domains, with the overarching goal to synthesize the neuroimaging literature of neural substrates of NSS in patients with a schizophrenia spectrum disorder.

## Results

### Study identification

**Fig. 1:**
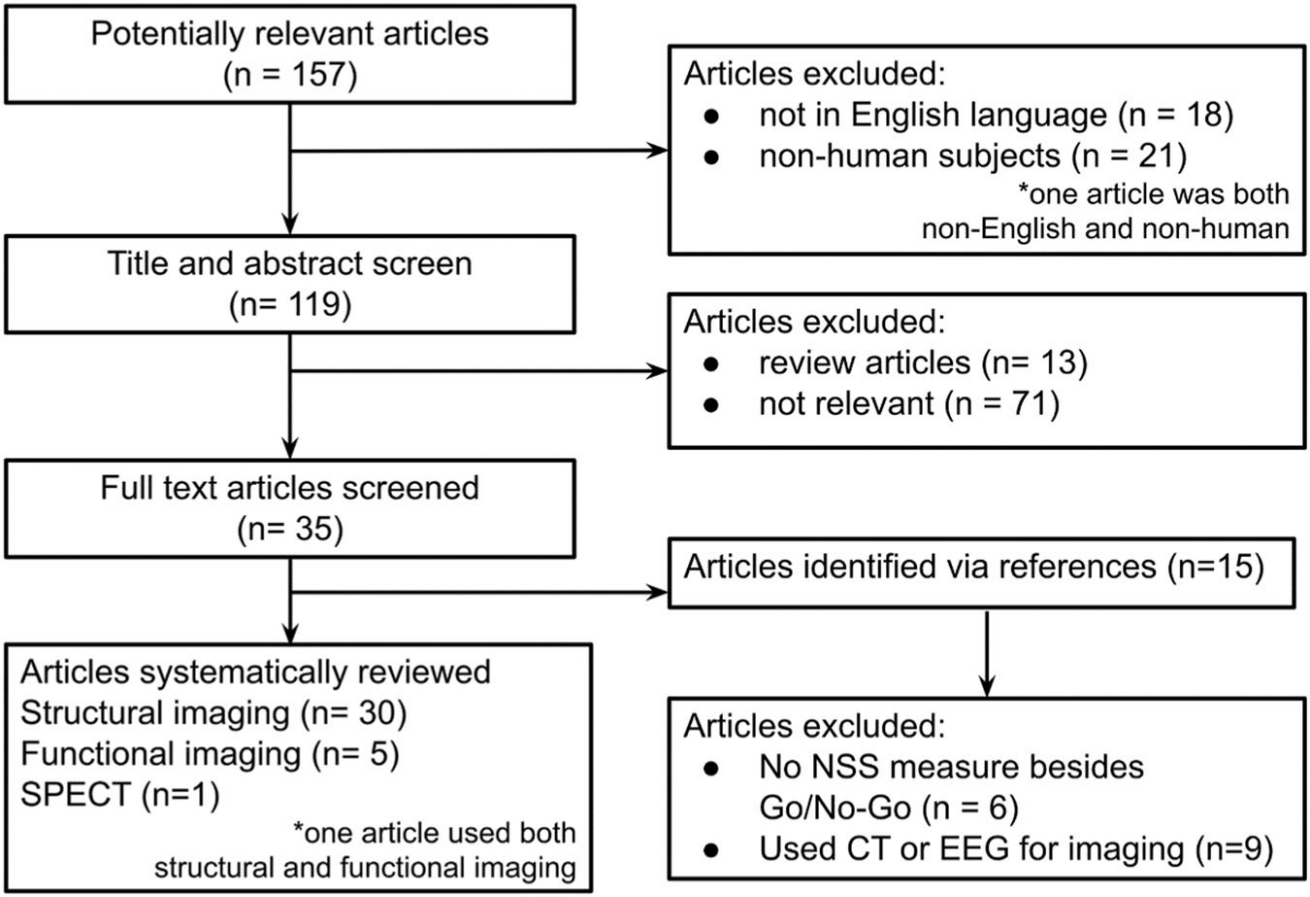
Outcomes at every level of our study identification process. Of the 157 potentially relevant articles, we included 35 in this systematic review (one study included both structural and functional imaging, and so was included in the *n* for both).

Figure 1 describes outcomes at each level of our study identification process. Of the 35 relevant articles we have included 29 structural MRI studies, 4 functional MRI, one study with both structural and functional MRI, and one molecular imaging study in this systematic review.

### Study characteristics

Sixteen of the structural studies, all of the functional studies, and the study featuring both methods compared patients to healthy controls, while thirteen structural and the one SPECT study only included patients. Of the twenty-one studies comparing patients to healthy controls, fourteen reported higher NSS scores in patients than in healthy controls. Patients’ illness stages in schizophrenia were chronic (*n* = 14), first episode (*n* = 15), and recent onset (*n* = 5); the SPECT study did not specify the patients’ illness stage.

### Structural MRI findings

A total of 30 structural imaging studies were included in the systematic review (Table 1a, b). The majority of studies quantified structural indices using T1 weighted imaging, one study used diffusion imaging, and one study used magnetization transfer imaging. Seventeen studies compared structural differences between patients and healthy controls (Table 1a); thirteen investigated patients only (Table 1b).

**Table 1:** (a) Comparative structural imaging studies. (b) Patient-only structural imaging studies.

Author	*n* (HC/PT)	Age, years (HC/PT, Mean ± SD)	Illness stage	Medication Status	NSS Scale	T	Imaging sequence	Structural assessment	Region of Interest	Group differences in NSS	Imaging findings
*(a) Comparative structural imaging studies.*											
Bottmer et al.^37^	18/37	25.65 ± 6.4/25.50 ± 2.4	First episode	Minimally treated	Heidelberg Scale	1.5	T1 weighted	Brain volume	Cerebellum	Not reported	Patients had lower cerebellar volumes compared to controls. NSS scores in patients negatively correlated with right cerebellar volumes.
Foong et al.^42^	30/25	35.03/37.28	Chronic	Medicated	Cambridge Neurological Inventory	1.5	Magnetization transfer imaging	manual Tracing	Corpus callosum, frontal, temporal, parietal and occipital white matter	NSS total scores patients >controls	White matter magnetization transfer ratio was lower in patients compared to controls. NSS scores were not correlated with the temporal magnetization transfer ratio in patients.
Galderisi et al.^75^^a^	13/13, 13^a^	NR/27.9 ± 7.6, 29 ± 7	Chronic	Medicated	Standardized neurological examination	1	T1 weighted	Expert grading, manual tracing	Slab covering the lateral ventricular system	Not reported	Patients had greater ventricle size and subarachnoid space to brain volume ratio compared to controls. No correlations between NSS scores and neuroimaging indices were performed.
Hirjak et al.^49^	37/37	34.08 ± 11.83/34.41 ± 11.00	Chronic	Medicated	Heidelberg Scale	3	Resting state fMRI and sMRI	Whole brain	mCCA+jICA	Not reported	NSS motor score was associated with a joint structural/functional group-discriminating component encompassing the frontocerebellar/frontoparietal networks in patients.
Hirjak et al.^39^	16/16	23.06 ± 4.2/23.68 ± 4.65	Recent onset schizophrenia	Medicated	Heidelberg Scale	3	T1 weighted	Cortical thickness, surface area, local gyrification index	Whole brain	NSS total scores patients >controls	Higher NSS total scores were correlated with lower cortical thickness in the paracentral lobule and precuneus. Higher NSS total scores were associated with greater cortical thickness in the middle temporal gyrus. Positive correlations between cortical thickness and higher NSS complex motor task scores were found in the middle temporal gyrus. Positive correlations were found between cortical thickness in the post-central gyrus and left right and spatial orientation scores. Higher NSS integrative function scores were positively correlated with cortical thickness in the middle temporal and supramarginal gyri and negatively correlated with cortical thickness in the paracentral and inferior parietal cortices and precuneus.
Hirjak et al.^39^	26/26	NR/23.38 ± 3.87	Recent onset schizophrenia	Medicated	Heidelberg Scale	3	T1 weighted	Voxel-based morphometry	Cerebellum, brain stem	NSS scores patients >controls	Higher NSS total scores were associated with lower cerebellum gray matter volume and lower brain stem white matter volume.
Hüttlova et al.^76^	23/24	30.83 ± 8.19/32.75 ± 9.67	Chronic	Medicated	NES	1.5	Diffusion weighted	Tractography	Corticospinal tract, superior cerebellar peduncle	NSS sequencing of complex motor tasks scores patients >controls	Patients with movement sequencing abnormalities showed lower fractional anisotropy as well as higher mean diffusivity and radial diffusivity in the corticospinal tract compared to controls. Patients without movement sequencing abnormalities did not differ in corticospinal tract diffusion measures. Sequencing of motor scores in patients was correlated with radial diffusivity and mean diffusivity in the superior cerebellar peduncle.
Kašpárek et al.^77^	19/37	23.8 ± 1.8/24.0 ± 4.6	First episode	Medicated	NES	1.5	T1 weighted	Voxel-based morphometry	Basal ganglia	not reported	Patients had greater gray matter concentrations in the pallidum and putamen compared to controls. Patients with movement sequencing abnormalities had lower putamen gray matter concentrations than patients without movement sequencing abnormalities.
Keshavan et al.^11^	18/17	23.94 ± 5.37/29.53 ± 8.46	First episode	Antipsychotic naïve	NES	1.5	T1 weighted	Manual tracing		NSS total scores patients >controls	In patients, a NES repetitive movement factor negatively correlated with caudate and cerebellum. The cognitive/ perceptual factor scores correlated negatively with caudate, cerebellum and heteromodal association cortex volumes.
Kong et al.^30^	20/18	52.18 ± 8.1/54.2 ± 8.7	Chronic	Medicated	Heidelberg Scale	3	T1 weighted	Cortical thickness	Whole brain	NSS total scores patients >controls	Patients showed a negative correlation between NSS total scores and cortical thickness in the middle frontal, inferior temporal, superior parietal, post-central and supramarginal cortices. Subscale scores showed negative correlations with cortical thickness. In controls, a negative correlation was found between NSS total scores and the rostral anterior cingulate cortex. Subscale scores showed a mix between positive and negative correlations with cortical thickness.
Kong et al.^34^	20/20	24.1 ± 3.5/25.6 ± 7.2	First episode	Medicated	Heidelberg Scale	1.5	T1 weighted	Voxel-based morphometry	Whole brain	NSS total scores patients >controls	Patients had significantly lower gray matter density in the cingulate, superior temporal gyrus and cerebellum compared to controls at baseline. After one year of follow up, patients with persistent NSS showed gray matter volume decline in the middle frontal gyrus, cingulate gyrus, claustrum and cerebellum. This decrease was less pronounced in those with decreasing NSS.
Sachdev et al.^78^	34/47	71.8 ± 7.4/62.1 ± 8.3/73.6 ± 9.7^b^	Chronic	Medicated	28 item standardized neurological examination	1.5	T1 weighted	Manual tracing	Whole brain		Patients had greater ventricle size, and more cortical atrophy in the anterior temporal and mid-parietal regions compared to controls. No correlations between NSS scores and brain volume measures were performed.
Szendi et al.^79^	13/13	29.3 ± 4.7/25.9 ± 5.4	Chronic	Medicated	NES	1	T1 weighted	Manual tracing	Straight gyrus, anterior cingulate, orbitofrontal cortex, middle frontal gyrus, hippocampus, third ventricle	NSS total scores patients >controls	Brain volumes did not differ between patients and controls in any region. No correlations between NSS scores and brain volume measures were performed.
Thomann et al.^38^	21/30	27.48 ± 4.86/27.73 ± 6.62	First episode	Medicated	Heidelberg Scale	1.5	T1 weighted	Manual tracing	Cerebellum	NSS total scores patients >controls	Cerebella of patients were significantly smaller compared to controls. This was most pronounced in the corpus medullae. Higher NSS scores in patients were correlated with lower cerebellum posterior lobe volumes.
Thomann et al.^38^	22/42	27.6 ± 3.5/27.7 ± 5.8	First episode	Medicated	Heidelberg Scale	1.5	MRI	Voxel-based morphometry	Whole brain	NSS total scores patients >controls	In patients, higher NSS scores were associated with lower gray matter density in the precentral, post-central, and lingual gyri, the insula, thalamus, caudate and cerebellum. Higher NSS scores were associated with lower white matter density in the inferior frontal gyrus, corpus callosum and cerebellum. In controls, higher NSS scores were correlated with lower gray matter density in the middle frontal and inferior frontal gyri.
Venkatasubramanian et al.^40^	27/30	27.4 ± 7.0/30.1 ± 8.3	Chronic	Antipsychotic naïve	NES	1.5	T1 weighted	Voxel-based morphometry	Whole brain	All NSS subscales patients >controls	Gray matter volumes were lower in the superior frontal and superior temporal gyri, as well as cuneus putamen, midbrain and cerebellum in patients compared to controls. Motor sequencing scores were negatively correlated with total gray matter volume, as well as gray matter volume in the left superior frontal, posterior cingulate, and middle temporal gyri, putamen, midbrain and cerebellum in patients.
Williams et al.^25^	40/40	NR/32 ± 6.8	Chronic	Medicated	Standardized neurological examination		Computed Tomography	Manual tracing	Ventricular-brain ratio	Not reported	Patients had a significantly higher ventricular-brain ratio than controls. The severity of NSS was not associated with the ventricular-brain ratio in patients.

Author	*n*	Age, years (Mean ± SD)	Illness stage	Medication Status	NSS Scale	T	Imaging sequence	Structural assessment	Region of Interest	Imaging findings
*(b) Patient-only structural imaging studies.*										
Bersani et al.^41^	29	31.72 ± 11.82	Chronic	Mixed	NES	1	T1 weighted	Manual tracing	Corpus callosum	Patients were divided into low and high NSS subgroups. No difference was found in total corpus callosum volume, length, or height. NES total scores and sequencing of complex motor acts subscale scores correlated with the width and area of the genu of the corpus callosum. Sequencing of complex motor acts scores also negatively correlated with the rostral corpus callosum body area.
Bersani et al.^26^	33	32.55 ± 8.37	Chronic	Medicated	NES	1	T1 weighted	Manual tracing	Interuncal index, third ventricle width, cerebellar vermis atrophy, Evans’ ratio	All MRI indices correlated with at least one item on the NES.
Ciufolini et al.^27^	66	27.65 ± 7.94	First episode	Medicated	NES	3	T1 weighted	Cortical thickness, surface area	Whole brain	Superior-parietal gyrus thickness was greater, but surface area in the lingual gyrus, superior temporal sulcus, and rostral middle frontal gyrus was lower in patients with high NSS scores compared to those with low NSS scores.
Dazzan et al.^35^	77	27.9 ± 8.7	First episode	Mixed	NES	1.5	T2 weighted	Gray and white matter volume	Whole brain	Putamen volume was negatively correlated with NSS scores in patients. Patients with high NSS scores had lower gray matter volume in putamen, pallidum and insula. No associations between white matter volumes and NSS were detected.
Gay et al.^80^	41	25.8 ± 6.0	First episode	Minimally treated	23 item standardized neurological examination	1.5	T1 weighted	Sulcal asymmetry, CSF volume	ACC	An interaction effect of ACC morphology, NSS scores, and handedness on cognitive control was found, indicating that early developmental abnormalities negatively affect performance on a cognitive control task.
Gay et al.^28^	44	26.0 ± 5.7	First episode	Mixed	23 item standardized neurological examination	1.5	T1 weighted	Cortical sulcation	Whole brain	Patients with NSS had a lower bilateral global sulcal index compared to those without NSS. No correlations between NSS total or subscale scores and global sulcal index were observed.
Heuser et al.^31^	102	27.2 ± 7.8	First episode	Medicated	Heidelberg Scale	1.5	T1 weighted	Voxel-based morphometry	Whole brain	NSS total scores were associated with reduced gray matter densities in the precentral and post-central gyri, the inferior parietal lobule and the inferior occipital gyrus. Motor coordination scores were negatively correlated with precentral gyrus, inferior parietal, and right cerebellar gray matter densities. Higher scores on the complex motor task subscale were associated with decreased densities in the middle frontal and superior frontal gyri, the insula, and precentral gyrus. Orientation scores were associated with reduced density in the medial frontal, inferior temporal and middle occipital gyri. Integrative function scores were correlated with lower gray matter density in the superior and inferior frontal gyri and medial frontal gyrus. Hard signs were associated with reduced density in the inferior occipital gyrus and cerebellum.
Hirjak et al.^39^	33	23.11 ± 4.24	Recent onset schizophrenia	Medicated	Heidelberg Scale	3	T1 weighted	Local gyrification index	Whole brain	NSS total scores were correlated with gyrification in the superior temporal, superior parietal and supramarginal cortices. Motor coordination scores were correlated with gyrification in the inferior temporal, superior parietal and supramarginal cortices. Complex motor task performance was associated with gyrification in the precentral, supramarginal and fusiform cortices. Integral function was associated with lateral occipital gyrification, and right/left and spatial performance was associated with gyrification in the precentral and middle temporal cortices as well as the precuneus.
Hirjak et al.^81^	28	23.21 ± 4.33	Recent onset schizophrenia	Medicated	Heidelberg Scale	3	T1 weighted	Cortical thickness	Whole brain	Higher NSS total scores were correlated with lower cortical thickness in the paracentral lobule and precuneus. Higher NSS total scores were associated with greater cortical thickness in the middle temporal gyrus. Positive correlations between cortical thickness and higher NSS complex motor task scores were found in the middle temporal gyrus. Positive correlations were found between cortical thickness in the post-central gyrus and left right and spatial orientation scores. Higher NSS integrative function scores were positively correlated with cortical thickness in the middle temporal and supramarginal gyri and negatively correlated with cortical thickness in the paracentral and inferior parietal cortices and precuneus.
Hirjak et al.^36^	21	22.6 ± 3.6	First episode	Medicated	Heidelberg Scale	3	T1 weighted	Brain volume, shape analysis	Brainstem	Higher NSS total, motor coordination, and complex motor scores are associated with lower brainstem volumes. This effect was seen predominantly in the pons and midbrain.
Hirjak et al.^82^	20	24.5 ± 4.4	Recent onset schizophrenia	Medicated	Heidelberg Scale	3	T1 weighted	Brain volume, shape analysis	Thalamus, caudate, putamen, and globus pallidus	Higher NSS total scores were associated with greater shrinkage in the thalamus and atrophy of the caudate in the anterior, posterior and medial part, as well as shape alterations of the pallidum. Higher motor coordination subscale scores were associated with lower thalamus and pallidum volume and volume alterations in the caudate.
Janssen et al.^43^	70	16.3 ± 1.7	First episode	Medicated	NES	1.5	T1 weighted	Voxel-based morphometry	Thalamus, caudate, hippocampus	Sensory integration scores correlated with lower anterior thalamus volumes. Sequencing of complex motor acts scores correlated with lower caudate volumes.
Mouchet-Mages et al.^33^	52	26.3 ± 7.09	First episode	Mixed	23 item standardized neurological examination	1.5	T1 weighted	Voxel-based morphometry	Whole brain	NSS total scores negatively correlated with dorsolateral prefrontal cortex density. Motor integration scores were negatively correlated with dorsolateral prefrontal cortex density. Motor coordination scores were positively correlated with thalamus gray matter density. Motor coordination scores were also negatively correlated with thalamus and cerebellum white matter densities.

A summary of the imaging studies assessing brain structural correlates of neurological soft signs in psychosis spectrum patients and healthy controls.

*n* number of subjects, *HC* healthy controls, *PT* patients, *SD* standard deviation, *T* Tesla, *CNI* Cambridge Neurological Inventory, *NES* Neurological Evaluation Scale, *SNE* standardized neurological examination, *VBM* voxel-based morphometry, *mCCA+jICA* multi-set canonical correlation analysis + joint independent component analysis, *NR* not reported.

^a^Galderisi et al. studied patients with simple schizophrenia vs. non-simple subtypes of schizophrenia; they did not report mean age of their healthy controls.

^b^Sachdev et al. studied patients with early onset (EOS) and late onset schizophrenia (LOS), so these ages are HC/EOS/LOS.

#### Associations between global NSS severity and structural imaging measures

To the best of our knowledge, the first neuroimaging study examining biological correlates of soft signs was conducted in 1985, using computed tomography to assess the ventricular-brain ratio. While an increase of ventricle size was evident in patients compared to controls, this was not associated with overall NSS severity^25^. In a later MRI study, ventricle volumes and lateral ventricular enlargement were found to be associated with NSS severity on at least one item of sensory integration, motor coordination, and sequencing of complex motor acts^26^, suggesting ventricular volume abnormalities (likely reflecting brain volume loss) may be relevant for NSS severity globally.

*Cortical gray matter thickness, surface, gyrification, and sulcation*. No differences in cortical thickness or surface area were reported in first episode patients with low versus high NSS burden^27^. While global cortical sulcus indices were lower in first episode patients with soft signs compared to those without, sulcation indices did not correlate with NSS severity^28^. In contrast, total NSS scores were found to be associated with gyrification in the orbitofrontal, superior temporal, superior parietal, and supramarginal cortices in recent onset schizophrenia patients^29^. In chronic patients, reduced cortical thickness in the middle frontal, inferior temporal, superior parietal, postcentral and supramarginal cortex was found to be associated with greater global NSS severity^30^.

*Cortical gray matter volumes or densities.* The largest of these studies was conducted in 102 inpatients diagnosed with a first psychotic episode. Global NSS severity was associated with decreased gray matter density in the precentral, postcentral, inferior parietal, and inferior occipital gyri^31^. The finding was a partial replication of an earlier study in first episode patients that reported an association between lower precentral and postcentral cortical gray matter density and NSS severity. Here, negative correlations were also observed with the lingual gyrus and insula^32^. Another study in first episode patients reported a relationship between global NSS severity and the dorsolateral prefrontal cortex volumes^33^. In a longitudinal study monitoring the temporal evolution of soft signs in first episode patients, Kong and colleagues report that those with persistent NSSs after one year of follow up showed more pronounced gray matter volume reductions in the frontal lobe, suggesting that persistence of NSSs may be a clinical marker of gray matter disease progression in first episode patients^34^.

*Subcortical and cerebellar volumes or densities.* Global soft sign severity in first episode psychosis patients was found to be associated with volume reductions in the putamen^35^, thalamus^32^, brain stem^36^, and cerebellum^37,38^. Similar brain signatures were also reported in recent onset schizophrenia patients where associations between NSSs and brain stem volumes^36,39^, as well as cerebellum volumes^11,32,34,37–40^ were detected. Consistent with this, Venkatasubramanian and colleagues reported an association between global soft sign severity and lower gray matter density in the caudate and cerebellum in antipsychotic medication-naïve patients with chronic schizophrenia^40^.

*White matter integrity.* Considerably fewer studies have assessed putative associations between white matter measures and soft signs. One study investigated the corpus callosum in chronic schizophrenia patients and found that total NSS scores were associated with the width of the genu of the corpus callosum, but did not find associations with any morphological features of the other six corpus callosum subregions^41^. In medicated first episode psychosis patients, higher NSS scores were associated with lower white matter density in the inferior frontal gyrus and the cerebellum^32^. In contrast, a large study conducted in first episode patients failed to reveal associations between white matter volumes and total NSS scores, or differences in white matter volumes between patients with a low or high NSS symptom burden^35^. This lack of associations between total NSS scores and white matter volumes was later replicated in a study focused on cerebello-thalamo-prefrontal structures in first episode patients^33^. Another study focused on the cerebellum reported a negative correlation between soft sign severity and white matter volume of the midbrain and cerebellum in recent onset schizophrenia patients^39^. The only magnetization transfer contrast study examining white matter integrity found a decrease in the temporal magnetization transfer ratio in patients compared to controls, suggestive of myelin or axonal disruption, but did not report an association with NSS total scores^42^.

#### Sensory integration and structural imaging measures

*Cortical gray matter thickness, surface, gyrification, and sulcation.* Studies examining associations of cortical thickness and/or cortical surface area in context of sensory integration deficits found that lower cortical thickness in the pre- and post-central gyrus was associated with greater symptom severity in first episode patients^27^. In the same patients, the cortical surface area was greater in the lateral occipital cortex in those who had a higher sensory integration scores compared to those with lower scores^27^. Gyrification in the lateral occipital cortex was associated with sensory integration scores in recent onset schizophrenia patients^29^. Interestingly, no associations between cortical thickness and sensory integration scores were reported in a small study of patients with chronic schizophrenia^30^.

*Cortical gray matter volumes or densities.* In first episode patients, higher sensory integration scores were found to be correlated with reduced gray matter density in the superior, medial, and inferior frontal gyri^31^, as well as reduced gray matter volume in the insula, inferior frontal, middle temporal, and lingual gyrus^35^, but others reported no relationships between sensory integration scores and gray matter volumes^33^.

*Subcortical volumes or densities.* In first episode patients, thalamus^35,43^, pallidum, and putamen volume reductions were associated with greater sensory integration scores^35^. Using a slightly different approach compared to the traditional subscales for the NSS, Keshavan and colleagues used a factor analysis method to examine structural imaging correlates of soft signs in a large group of antipsychotic medication-naïve first episode psychosis patients. The factor that was comprised of complex tasks that were cognitively demanding and involved sensory processing showed negative correlations with caudate and cerebellum volumes^11^.

*White matter integrity.* First episode psychosis patients with high sensory integration scores had greater left internal capsule volumes compared to those with low sensory integration scores^35^. In chronic patients, the width of the splenium of the corpus callosum was found to be greater in those with low sensory integration scores compared to those with high sensory integration scores^41^.

#### Motor coordination and structural imaging measures

*Cortical gray matter thickness, surface, gyrification, and sulcation.* Studies examining associations of cortical thickness and/or cortical surface area in the context of motor coordination subscale scores found that first episode patients with higher scores showed reduced cortical thickness in the inferior parietal, superior temporal, and fusiform gyrus, as well as reduced surface area in the superior frontal and orbitofrontal gyrus^27^. In recent onset schizophrenia patients, higher motor coordination scores were associated with reduced cortical thickness in the precentral gyrus^29^, and in chronic patients, this association was seen in the inferior frontal and postcentral gyri^30^. Gyrification in the inferior temporal and supramarginal cortices were associated with motor coordination scores in recent onset schizophrenia patients^29^.

*Cortical gray matter volumes or densities.* In first episode patients, greater motor coordination scores were associated with decreased gray matter density in the precentral gyrus and inferior parietal lobe^31^.

*Subcortical volumes or densities.* In first episode patients, thalamus volumes were positively correlated with motor coordination scores^33^. In contrast, motor coordination abnormalities were found to be associated with volume reductions in the caudate^11^, putamen^35^, brain stem^36^, cerebellar volumes^11^, cerebellar gray matter^31^, and cerebellar white matter^33^.

*White matter integrity.* In first episode patients, one group found an inverse correlation between motor coordination scores and corpus callosum volumes^32^, whereas others found that patients with high motor coordination scores had greater left internal capsule volumes compared to those with low motor coordination scores^35^.

#### Sequencing of complex motor tasks and structural imaging measures

*Cortical gray matter thickness, surface, gyrification and sulcation.* No relationships between sequencing of complex motor task scores and cortical thickness or surface area were reported^27^. On the other hand, reduced gyrification in the right temporal pole^29^ and supramarginal and fusiform cortices^29^ were found to be associated with complex motor task scores in recent onset schizophrenia patients.

*Cortical gray matter volumes or densities.* In first episode patients, decreased gray matter densities in the superior and middle frontal gyri^31,40^, precentral gyrus^31^, insula^31^, and posterior cingulate cortex^40^ were found to be associated with greater sequencing of complex motor tasks scores. However, others report no such associations between cortical gray matter volumes and motor sequencing scores^35^.

*Subcortical volumes or densities.* In terms of subcortical and cerebellar regions, scores on the complex motor task subscale were associated with volume reductions in the putamen,^40,44^ midbrain^40^, brain stem^36^, and cerebellum^38,40^.

*White matter integrity.* One study investigated the corpus callosum in chronic schizophrenia patients and found that scores on the sequencing of complex motor acts scale were positively correlated with the genu width and genu area, and negatively correlated with the corpus callosum rostral body area^41^. A tractography study of the corticospinal tract and superior cerebellar peduncle, which had both tracts involved in motor sequencing, found that patients with sequencing abnormalities showed reduced fractional anisotropy in the superior cerebellar peduncle compared to patients without sequencing abnormalities and healthy controls. In contrast, only patients without sequencing abnormalities had reduced corticospinal tract fractional anisotropy and increased radial diffusivity. Both radial and mean diffusivity in the superior cerebellar peduncle were correlated with severity of sequencing abnormality scores in the entire group, suggesting that white matter integrity is involved in NSSs^45^.

### Functional MRI findings

Of the five functional studies included here, four utilized task fMRI, and one was a combined resting state fMRI and structural MRI study (Table 2). All studies were conducted in patients who were on antipsychotic medications at the time of the assessment, and all but one focused on patients in the chronic disease stage.

**Table 2:** Functional imaging studies.

Author	*n* (HC/Pt)	Age, years (HC/PT, Mean ± SD)	Illness stage	Medication status	NSS Scale	T	Imaging sequence	Region of Interest	Analysis type	Group differences in NSS	Imaging findings
Chan et al.^46^	14/14	21.71 ± 3.81/20.08 ± 3.38	First Episode	Medicated	CNI	3	FEP, PT, PS task fMRI	Whole brain	BOLD response, PPI	CNI total scores did not differ between groups	Activation in left frontal and parietal regions was lower during PT in patients compared to controls. Greater task complexity was associated with greater activation in middle frontal regions in controls but not patients.
Hirjak et al.^49^	37/37	34.08 ± 11.83/34.41 ± 11.00	Chronic	Medicated	Heidelberg Scale	3	Resting state fMRI and sMRI	Whole brain	mCCA+jICA	Not reported	NSS motor score was associated with a joint structural/functional group-discriminating component encompassing the frontocerebellar/frontoparietal networks in patients.
Kasparek et al.^44^	24/24	31.8 ± 9.2/32.8 ± 9.7	Chronic	Medicated	NES	1.5	Finger tapping task, finger-thumb opposition task fMRI	Whole brain	BOLD response, functional connectivity (seed based)	Patients had higher NES scores compared to controls	Abnormal cortico-cerebellar functional connectivity during the execution of a motor task is linked with movement sequencing abnormalities in patients.
Schröder et al.^83^	7/10	26.0 ± 2.45/29.2 ± 4.64	Chronic	Medicated	Heidelberg Scale	1.5	Finger-thumb opposition task fMRI	Central and sensori-motor cortex	BOLD response	Not reported	Activation in the sensorimotor areas and supplementary motor cortex is diminished in patients.
Zemankova et al.^47^	24/24	31.75 ± 9.20/32.75 ± 9.67	Chronic	Medicated	NES	1.5	Finger tapping task, finger-thumb opposition task fMRI	Whole brain	BOLD response, PPI, Granger causality	Patients more frequently showed impairment in sequencing of complex motor acts	Hyperactivation of the parietal cortex is linked to motor symptoms. Aberrant frontoparietal connectivity was more pronounced in those with soft signs compared to those without.

A summary of the findings of the functional imaging studies reviewed.

*n* number of subjects, *HC* healthy controls, *Pt* patients, *T* Tesla, *CNI* Cambridge Neurological Inventory, *NES* Neurological Evaluation Scale, *FEP* Fist-Edge-Palm Task, *PT* palm-tapping task, *PS* pronation/supination task, *mCCA+jICA* multi-set canonical correlation analysis + joint independent component analysis.

The four studies that assessed brain function during task performance all used motor tasks. Both Chan et al.^46^ and Zemankova et al.^47^ reported aberrant task activation in frontoparietal regions. In first episode patients, reduced task activation and functional connectivity were reported, and patients failed to increase frontal activation with increasing task difficulty^46^. In contrast, in chronic patients an increase in frontoparietal task activation was noted; this overactivation was interpreted as evidence of a compensatory strategy for patients to achieve adequate motor performance. Complementary frontoparietal network functional connectivity analysis further suggested that an interhemispheric cortical inhibition deficit which was most pronounced in patients who had movement sequencing difficulties^47^. However, others did not find evidence of aberrant task activation in patients with chronic schizophrenia, regardless of whether patients did or did not display sequencing difficulties^44^.

Nonetheless, movement sequencing abnormalities in patients in this study were found to be linked to abnormal cortico-cerebellar functional connectivity. In a region of interest analysis examining activation of the sensorimotor cortex and supplemental motor cortex, Schröder et al. found reduced task activation in both regions in patients with chronic schizophrenia. The extent of task activation between the sensorimotor cortex and the supplemental motor cortex was correlated, suggesting a functional coupling of these brain regions. The authors concluded that dysfunction in these brain regions may contribute to NSS severity^48^.

The only relevant study assessing brain function at rest did so in a multimodal approach, also considering gray matter structural integrity in the analysis^49^. Interestingly, NSS motor scores were found to be associated with a joint structural/functional group-discriminating component encompassing frontoparietal and frontocerebellar networks in patients.

Taken together, aberrant function in motor, frontoparietal and cerebellar networks may be associated with NSS in patients with a schizophrenia spectrum disorder.

### Molecular imaging findings

One molecular imaging study using SPECT was included in the systematic review. This report examined dopamine D2 receptor binding in a group of 23 antipsychotic medication-naïve schizophrenia patients. Importantly, they found that NSSs are already present in patients who have never been medicated, and that these may decrease in severity after antipsychotic treatment. However, no direct relationships between NSS severity and baseline D2 receptor binding or antipsychotic medication related change in D2 binding were detected^50^.

## Discussion

Here, we performed a systematic review of the neuroimaging literature of NSS in patients with a schizophrenia spectrum disorder with the goal to synthesize the existing literature. We included studies which have a neuroimaging component and a standardized NSS measure. Studies consistently implicate the basal ganglia and cerebellum as structural substrates of NSS and suggest that structural alterations in somatomotor and somatosensory regions, as well as areas involved in visual processing and spatial orientation, may underlie NSS in psychosis spectrum disorders. Additionally, dysfunction of frontoparietal and cerebellar networks has been implicated in the pathophysiology of NSS in several studies. In contrast, the limited published literature on white matter volume deficits and dopamine D2 receptor dysfunction suggest that these markers may not play a primary role in the development of NSS, though those findings clearly warrant replication.

Our results are consistent with findings from a previous meta-analysis^24^ that included six structural and fifteen functional neuroimaging studies (the ALE meta-analysis included functional studies that used a go/no-go task in lieu of measurements designed to quantitatively assess NSSs across domains). Despite the difference in inclusion criteria, they identified a number of overlapping brain regions, including frontal regions, basal ganglia, and the cerebellum, that were associated with soft signs. Our findings not only confirm prior meta-analytic data, but they also expand efforts for synthesis of the literature by attempting to dissect the neurobiological correlates of NSS with respect to individual domains of soft signs. Interestingly, studies reported associations between NSS and basal ganglia structural integrity across domains. Only a few findings regarding involvement of a specific brain region in an individual domain of NSSs are replicated at this point. Sensory integration scores were found to be associated with lateral occipital cortex integrity in two studies^27,29^ and the association of motor coordination scores and precentral as well as inferior parietal gray matter integrity was reported more than once^31,32^. The lack of replication in structural correlates of NSS in specific domains may be attributable to a number of factors, including the small number of studies that investigated specific neurobiological signatures of NSS within a specific domain, the different instruments used to assess NSS, the heterogeneity in sample characteristics, and the variety of image analysis approaches, to name just a few. Similarly, the limited number of functional imaging studies conducted prevents us from making definitive conclusions on the functional circuitry of NSS.

In conclusion, a number of scientific questions around the central theme of neurobiological signatures of soft signs in psychosis spectrum disorders remain unanswered. For example, the literature is not consistent as to whether soft signs change across time in patients with psychosis spectrum disorders^51–53^. A meta-analysis suggests that the temporal evolution of soft signs may be a function of the illness course^16^, and different brain structures may differentially contribute to soft signs at different disease stages^54^, but the underlying pathophysiology remains unclear. To our knowledge, only two longitudinal studies examining NSSs also included an imaging component. One included 20 first episode schizophrenia patients who were followed over one year. While a significant decrease in NSS scores was found in the overall group, the subgroup without reductions in NSS showed pronounced gray matter reductions in the frontal lobe, cingulate gyrus, and cerebellum, suggesting that persistent NSS may be associated with progressive brain changes^34^. Another study assessed NSS severity in antipsychotic-naïve patients at baseline and after a variable length of follow-up, with imaging done at baseline. Here, smaller left dorsolateral prefrontal lobe volume at baseline predicted greater negative symptoms and poorer functional outcome on follow-up, but there was no significant change in NSS from baseline to follow-up^55^. Clearly, well-powered, longitudinal studies are needed to comprehensively characterize the neurobiological correlates of temporal changes in NSS, especially given its potential for biomarker development.

Similarly, the extent to which antipsychotic medications confound imaging findings with respect to soft signs has not been determined. The majority of studies investigated patients who were medicated at the time of the assessment, which can influence measurements on two levels. First, antipsychotic medications could affect the extent to which soft signs are behaviorally expressed^56,57^. NSS severity may improve with treatment and over time^58^. On the other hand, a study in children and adolescents at ultra-high risk (UHR) for psychosis found that antipsychotic treatment does not affect severity of NSS^59^, and another found no relationship between current antipsychotic dosage and NSS severity^60^. Second, medications do affect brain structure, function, and neurochemistry^61–71^. In this context, pharmacological challenge studies could help determine to what extent antipsychotic medications play a role in the phenotypic and neurobiological expression of NSSs.

This review must be considered in the context of some limitations. It is important to note that we did not use quantitative meta-analytic techniques, in part because the heterogeneity in data acquisition and analytic techniques was significant, which would make it difficult to make meaningful quantitative comparisons. Most studies had a relatively small sample size, and the majority of studies were conducted in patients who were treated with an antipsychotic medication. While there have been a relatively large number of studies conducted in first-episode psychosis patients, only two were performed in antipsychotic medication-naïve patients, which is a clear limitation. Next, it is important to note that the vast majority of studies assessed gray matter structure, but only few studies leveraged other imaging methods such as diffusion weighted imaging, or molecular imaging. Because of this, it remains unclear if white matter integrity deficits or neurometabolic alterations contribute to NSS in the illness. Another limitation is the use of various soft sign instruments in the reviewed studies, which could have contributed to the limited replications of findings. Developing a gold standard instrument for NSS assessments could be helpful in this regard. Future studies filling these important gaps in the literature will be helpful in advancing our mechanistic understanding of NSS and in assessing the potential of clinical and neurobiological markers of NSS in patients suffering from a psychosis spectrum disorder in the development neuroimaging biomarkers^72^.

## Methods

### Eligibility criteria

We included studies that assessed NSSs and obtained neuroimaging data in patients with a schizophrenia spectrum disorder published up to June 2020 (date of last literature search: June 15, 2020). We excluded the following:

- review articles
- studies published in languages other than English
- non-human studies
- studies that did not include a neuroimaging component
- studies that did not include a specific instrument designed to measure NSSs
- studies with fewer than 10 subjects
- studies expressly including subjects with neurological or genetic diseases, or intellectual disabilities

We did not exclude any studies based on the age of their study sample.

### Literature search

Using legacy PubMed, we used the following search term: “(neurological evaluation scale OR soft signs OR NES) AND (schizophrenia OR psychosis OR schizoaffective) AND (neuroimaging OR MRI OR Spectroscopy OR functional OR structural MRI OR white matter OR diffusion OR DTI OR gyrification OR cortical thickness OR neurometabolite OR functional connectivity OR network OR PET OR SPECT)”. Reference lists of those studies, as well as the initial results which were review articles or meta-analyses, were inspected for additional relevant publications.

### Study selection

After removing duplicate articles, GDS and NVK screened titles and abstracts to exclude irrelevant articles. Both authors applied eligibility criteria, and a list of eligible full text articles was developed through consensus. Full text articles that were not immediately available through the university library were requested, and all requests were fulfilled.

### Data extraction

We extracted the following information from each study: name of first author, year of publication, number of participants per diagnostic category, average age of patients, illness duration and medication status of patients, instrument used to quantify NSSs, magnetic field strength, neuroimaging modality, data analysis type, and reported main study outcomes for NSS and neuroimaging data, and tabulated findings separately for structural and functional neuroimaging studies.

To complement the high-level summary of each study presented in the table, we also provide a narrative description of main results in the Results section. The section for structural MRI findings was thematically arranged as follows: “cortical gray matter thickness, surface, gyrification, and sulcation”, “cortical gray matter volumes or densities”, “subcortical and cerebellar volumes or densities”, and “white matter integrity”, for which findings will be separately discussed with respect to global soft signs, sensory integration, motor coordination, and sequencing of complex motor task scores. These domains were chosen because they have been reliably implicated as subscales in different factor analyses of NSS battery items^2,73,74^. Because the number of functional and molecular imaging studies were much smaller, such a structure was not warranted, and we provided a narrative review summarizing major findings and highlighting similarities across studies instead.
